# Type 1 diabetes patients increase CXCR4^+^ and CXCR7^+^ haematopoietic and endothelial progenitor cells with exercise, but the response is attenuated

**DOI:** 10.1038/s41598-021-93886-2

**Published:** 2021-07-15

**Authors:** Guy S. Taylor, Andy Shaw, Kieran Smith, Tess E. Capper, Jadine H. Scragg, Michael Cronin, Ayat Bashir, Anneliese Flatt, Matthew D. Campbell, Emma J. Stevenson, James A. Shaw, Mark Ross, Daniel J. West

**Affiliations:** 1 Population Health Sciences Institute, Newcastle University, Newcastle upon Tyne, UK; 2 Translational and Clinical Research Institute, Newcastle University, Newcastle upon Tyne, UK; 3 Centre for Public Health, Queen’s University Belfast, Belfast, UK; 4 Nuffield Department of Primary Care Health Sciences, University of Oxford, Oxford, UK; 5 Faculty of Health Sciences and Wellbeing, University of Sunderland, Sunderland, UK; 6 Leeds Institute of Cardiovascular and Metabolic Medicine, University of Leeds, Leeds, UK; 7 School of Applied Sciences, Edinburgh Napier University, Edinburgh, UK

**Keywords:** Stem cells, Cardiology, Diseases, Endocrinology, Medical research, Risk factors

## Abstract

Exercise mobilizes angiogenic cells, which stimulate vascular repair. However, limited research suggests exercise-induced increase of endothelial progenitor cell (EPCs) is completely lacking in type 1 diabetes (T1D). Clarification, along with investigating how T1D influences exercise-induced increases of other angiogenic cells (hematopoietic progenitor cells; HPCs) and cell surface expression of chemokine receptor 4 (CXCR4) and 7 (CXCR7), is needed. Thirty T1D patients and 30 matched non-diabetes controls completed 45 min of incline walking. Circulating HPCs (CD34^+^, CD34^+^CD45^dim^) and EPCs (CD34^+^VEGFR2^+^, CD34^+^CD45^dim^VEGFR2^+^), and subsequent expression of CXCR4 and CXCR7, were enumerated by flow cytometry at rest and post-exercise. Counts of HPCs, EPCs and expression of CXCR4 and CXCR7 were significantly lower at rest in the T1D group. In both groups, exercise increased circulating angiogenic cells. However, increases was largely attenuated in the T1D group, up to 55% lower, with CD34^+^ (331 ± 437 Δcells/mL vs. 734 ± 876 Δcells/mL *p* = 0.048), CD34^+^VEGFR2^+^ (171 ± 342 Δcells/mL vs. 303 ± 267 Δcells/mL, *p* = 0.006) and CD34^+^VEGFR2^+^CXCR4^+^ (126 ± 242 Δcells/mL vs. 218 ± 217 Δcells/mL, *p* = 0.040) significantly lower. Exercise-induced increases of angiogenic cells is possible in T1D patients, albeit attenuated compared to controls. Decreased mobilization likely results in reduced migration to, and repair of, vascular damage, potentially limiting the cardiovascular benefits of exercise.

Trial registration: ISRCTN63739203.

## Introduction

Endothelial progenitor cells (EPCs), first discovered in 1997, are mononuclear cells which have the potential to stimulate vascular repair^1^. Evidence demonstrates that these cells can differentiate into endothelial cells in vitro^1,2^, incorporate into sites of angiogenesis in vivo^3,4^ and exert proangiogenic abilities via paracrine action^2^. First identified as cells in peripheral blood expressing CD34, a marker of haematopoiesis^5^, these precursor cells are now known as haematopoietic stem/progenitor cells (HPC). It is suggested that a more focused phenotype that includes endothelial markers, such as VEGFR2, identifies a sub-population that can differentiate into endothelial cells and therefor are true EPCs^6^.

The number and function of both HPCs and EPCs are clinically relevant, with lower concentrations associated with endothelial dysfunction^7^ and a greater risk of cardiovascular events and mortality^8,9^. Within individuals with type 1 diabetes, most^10–13^, but not all studies^14^, have found reduced circulating numbers of HPCs and EPCs compared to matched non-diabetes controls. In combination with hyperglycemia and glucose fluctuations, it is possible that dysfunctional HPCs and EPCs contribute to increased vascular damage^15,16^ and progression of micro and macrovascular complications^17^, with individuals with type 1 diabetes having a two- to eightfold increase in mortality rates compared with the general population largely due to cardiovascular diseases (CVD)^18–20^. Whilst improved glycemic control is associated with reduced development of CVD^21^, incidence remains elevated even in individuals who have successfully addressed modifiable risk factors^18^.

In healthy individuals, acute exercise can mobilize both HPCs and EPCs into circulation, and improve their angiogenic function^22–24^. However, exercise-induced increases of EPCs appears attenuated in those with chronic diseases^25,26^, which may partially explain the increased CVD risk in these populations. Indeed, increased pre-operative exercise-induced mobilization of EPCs is correlated with reduced post-operative complications after major thoracic surgery^27^, while HPCs response to exercise was a stronger predictor of myocardial ischemia and mortality than resting circulating count in patients with coronary artery disease over a subsequent 3 year period^28^. Insight into the ability of these cells to respond to a stimulus, such as exercise, migrating into circulation and homing to ischemic tissue can be measured by the surface expression of chemokine (C-X-C motif) receptor 4 (CXCR4) and 7 (CXCR7)^29,30^, although evidence on the influence of type 1 diabetes is lacking. Within other chronic diseases, diminished number of CD34^+^CXCR4^+^ cells may be a better predictor of mortality than CD34^+^ cells alone^9^, while the expression of CXCR7 has been linked to cell survival in diabetic condition in vitro*,* although limited evidence exists in human studies^30^.

While mobilisation of HPCs and EPCs appears attenuated to direct stimulation in both type 1 and 2 diabetes^31,32^, and exercise-induced increases appears attenuated in type 2 diabetes^26^, there is limited information in Type 1 diabetes, a vastly different disease. Type 1 diabetes patients are typically not obese, tend to be diagnosed at an early age (if not childhood), and generally live much more active lives with higher levels of cardio-respiratory fitness^33^, albeit slightly lower than the general non-diabetes general public^34^. At present, the two studies that have had investigated EPC mobilization with acute exercise in individuals with type 1 diabetes have found total lack of mobilisation^35,36^. However, as previous studies have measured EPCs as a percentage of circulating mononuclear cells, where any mobilization is likely masked by increases in overall leucocyte counts with exercise^37^, they failed to capture the expected post-exercise mobilization in the non-diabetes controls.

Thus, due to the increased risk of vascular complications in this disease, this study aimed to definitely explore whether exercise-induced increases of HPCs and EPCs is possible for people with type 1 diabetes. Additionally, this study aimed to explore how type 1 diabetes influences deeper phenotypes of angiogenic cells, including not previously measured cell surface expression of key chemotactic receptors CXCR4 and CXCR7, comparing to age-, sex-, fitness- and BMI- matched controls at rest and during exercise-induced mobilisation. We hypothesized that the type 1 diabetes group will have reduced resting and exercise-induced increases of HPCs and EPCs compared to healthy controls.

## Methods

### Participants

Participants were recruited from the Newcastle Diabetes Centre and Newcastle University. Participants with type 1 diabetes had a confirmed clinical diagnosis; age 18–65 years with a diabetes duration ≥ 3 years; HbA1c < 86 mmol/mol (10.0%); and absence of diabetes-related complications apart from non-proliferating retinopathy. Eligibility criteria for the non-diabetes participants comprised being aged between 18 and 65 years, non-smoker, and free from any history of chronic diseases.

All participants provided written informed consent and the study was approved by the NHS HRA North East Tyne & Wear South Research Ethics and Newcastle University Ethics Committees (code:16/NE/0192, registry:ISRCTN63739203). All methods were performed in accordance with the relevant guidelines and regulations.

### Screening visit

All participants attended the Newcastle NIHR Clinical Research Facility (CRF) on two occasions. Firstly, a screening visit to determine eligibility, medical assessment and peak oxygen uptake ($\dot{V}{\text{O}}_{{2{\text{peak}}}}$). Participant height, body mass (Seca 220 stadiometer / Seca 889 scales, Seca, Germany) and medical history were taken. Participants underwent a modified 12-lead resting and exercising electrocardiogram to screen for cardiac abnormalities. Eligible participants completed a maximal graded exercise treadmill (Valiant 2 CPET, Lode, Groningen, Netherlands) test using the Bruce protocol^38^ to determine $\dot{V}{\text{O}}_{{2{\text{peak}}}}$. Glucose levels in participants with type 1 diabetes were managed as per the guidance of Riddell et al.^39^.

### Main trial visit

Participants attended the CRF at least 7 days after the initial screening. Individuals arrived at the exercise lab at ~ 8.30am after an overnight fast, having been instructed to avoid structured exercise in the 48 h preceding the visit.

The participants with type 1 diabetes maintained their normal basal insulin regimen. If they experienced a hypoglycemic event overnight prior to the study visit, the visit was reorganised. If blood glucose on waking was > 10 mmol/L, they were instructed to have a small corrective bolus of rapid-acting insulin (≤ 2 units).

Upon arrival, the non-dominant arm of each participant was cannulated and resting (baseline) blood samples were drawn. The initial 4 mL drawn was discarded to avoid contamination of mature circulating endothelial cells with cells released from the punctured vein during the cannulation. One 10 mL EDTA vacutainer (Becton, Dickinson and Company, New Jersey, USA) was collected at baseline and, immediately post-exercise. An additional 4 mL EDTA Vacutainer was drawn at baseline for analysis of HbA1c at the Newcastle Clinical Laboratory. Capillary blood was collected at all-time points and analysed by a HemoControl analyser (EKF, Cardiff, UK) to determine haematocrit and haemoglobin concentration.

Participants consumed a 30 g carbohydrate snack (Belvita, Mondelēz International, USA) immediately after baseline blood draws and remained rested for 20 min. Participants walked on an incline for 45 min at 60% $\dot{V}{\text{O}}_{{2{\text{peak}}}}$ at a comfortable stride length (8.06 ± 5.09% at 4.30 ± 0.47 kph). Participants’ treadmill velocity and gradient were calculated using $\dot{V}{\text{O}}_{2}$, velocity, and gradient data from the preliminary $\dot{V}{\text{O}}_{{2{\text{peak}}}}$ test^40^. Breath-by-breath respiratory parameters (Metalyzer 3B-R3 CPET, Cortex, Leipzig, Germany) were continuously recorded throughout, with gradient adjusted at 10 and 30 min if $\dot{V}{\text{O}}_{2}$ was > 10% different than target $\dot{V}{\text{O}}_{2}$. Participants with type 1 diabetes had a target capillary blood glucose > 7 mmol/L for the duration of the exercise with 6 individuals given 10 g of additional carbohydrates, administered via a glucose drink.

Upon completion of the exercise, venous blood samples were immediately drawn from the cannula. Participants rested for 60 min before another venous blood sample was drawn and being discharged from the CRF if capillary blood glucose concentration > 3.9 mmol/L (70 mg/dL).

### Flow cytometry enumeration of hematopoietic and endothelial progenitor cells

HPCs and EPCs were quantified on a flow cytometer (BD LSRFortessa X20; BD Biosciences, USA) within 4 h of blood draw^6^. Briefly, 200 µL of whole peripheral blood collected in EDTA was incubated with 10 µL anti-CD34 FITC, 10 µL anti-VEGFR2 APC, 10 µL anti-CD45 BV421 (BioLegend, San Diego, CA, USA), 10 µL anti-CXCR4 APC Cy7, and 10 µL anti-CXCR7 PE (BioLegend, San Diego, CA, USA) in a BD Trucount (BD Biosciences, USA) tube at 4 °C for 30 min in the dark. Four mL of red blood cell lysis buffer (BD Pharm LyseTM, BD Biosciences, United Kingdom) was added and left to incubate for a further 30 min at 4 °C in the dark before enumeration by flow cytometry. The samples were vortexed at low speed to resuspend beads and reduce cell aggregation. Samples were analysed for 45 min or until 500,000 CD45^+^ events had been enumerated, whichever occurred first. The LSRFortessa was equipped with a blue, yellow/green, red, violet and ultra violet lasers (488 nm, 561 nm, 635 nm, 405 nm and 355 nm wavelengths, respectively).

**Figure 1:**
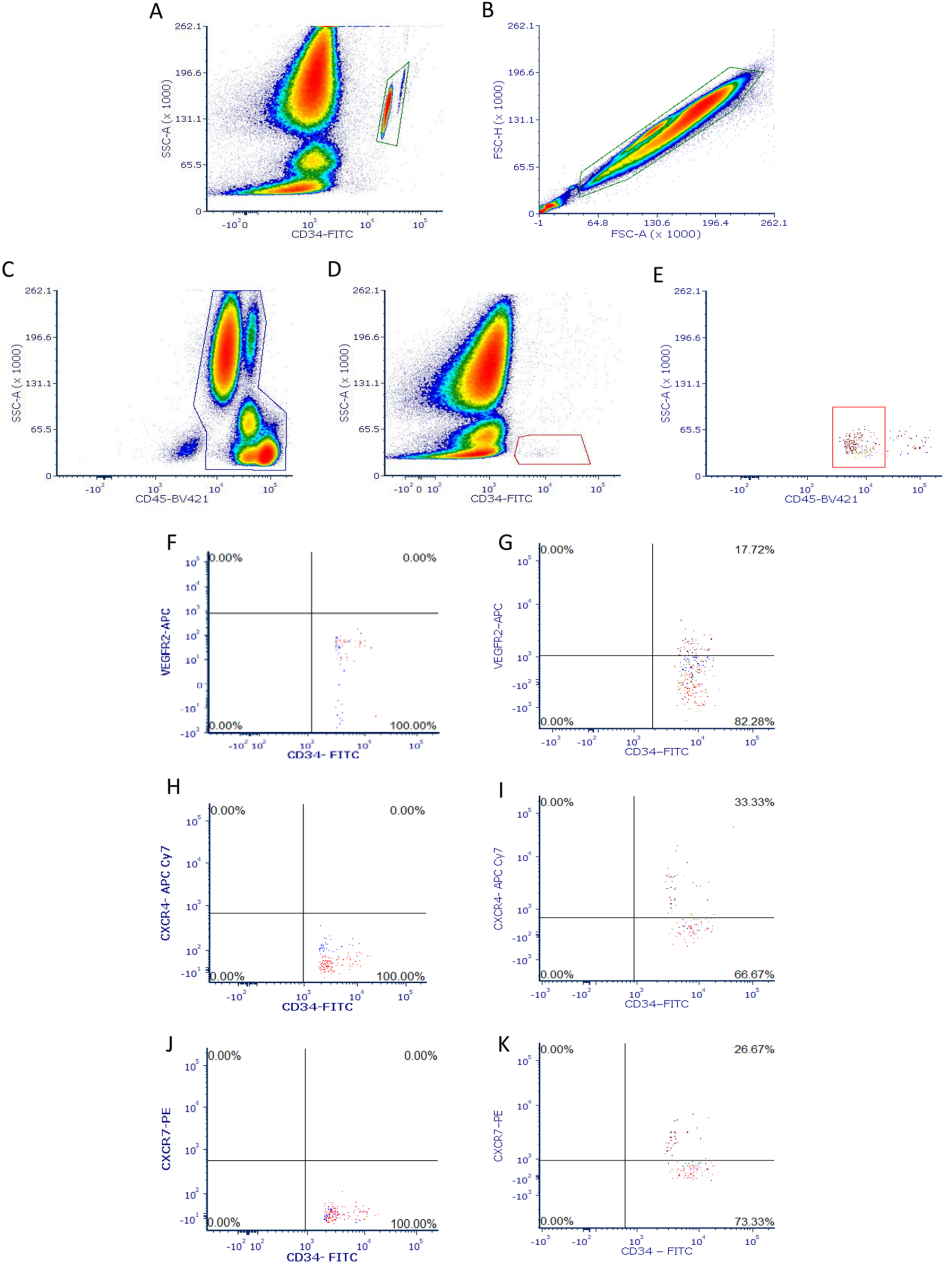
Enumeration of HCPs and EPCs by flow cytometry. (**A**) Gating of the fluorescent beads from the Trucount Tubes to determine absolute cell count. (**B**) Forward scatter height versus forward scatter area density plot for gating doublet exclusion. (**C**) Gating of CD45^+^ mononuclear cells. (**D**) Identification of CD45^+^ cells expressing CD34^+^ with low side scatter (CD34^+^ cells). (**E**) Gating of low expression of CD45^+^ (CD34^+^CD45^dim^ cells). (**F**) Negative controls for the identification the gating of positive VEGFR2^+^ events. (**G**) Identification of VEGFR2^+^ on CD34^+^ or CD34^+^CD45^dim^ cells. (**H**) Negative controls for the identification the gating of positive CXCR4 events. (**I**) Identification of CXCR4 cell surface expression upon all HPC and EPCs phenotypes. (**J**) Negative controls for the identification the gating of positive CXCR7 events. (**K**) Identification of CXCR7 cell surface expression upon all HPC and EPCs phenotypes.

Compensation using BD CompBead (BD Biosciences, USA), was performed prior to collecting each participant’s data to correct for any spectral overlap. Due to highly unreliable nature of isotype controls in rare event analysis^6^, positive (VEGFR2) and negative (VEGFR2, CXCR4, CXCR7) control samples were used to help determine the gating of positive events by histogram and dot plot (Fig. 1F,H,J). Between samples, FACS clean (BD Biosciences, USA) and deionized water was used to decontaminate the flow cytometer for 5 min.

Following data acquisition, flow cytometry files were analysed using FCS Express 7 (De Novo, California, USA). Counts of HPC and EPC numbers were converted to cells/mL using BD Trucount, with the number of positive cell events divided by the number of Trucount bead event, and then multiplying by the known total BD Trucount bead concentration. Haematocrit and haemoglobin concentration measures were used to adjust absolute cell counts changes in blood volume using the Dill and Costill method^41^. Instead of presenting as a proportion of total events enumerated by flow cytometry, a valid methodology for the measuring of rare cells at rest^6^, the use of Trucount tubes permits the acquisition of absolute cell counts of cells, and allows the exact changes in response to a stimulus to be measured. As overall leucocyte counts acutely increase with exercise^37^, any changes in rare cells are likely masked or hidden when measure as a percentage of total events.

The gating strategies for enumeration of the HPCs (CD34^+^, CD34^+^CD45^dim^)^5^ and EPCs (CD34^+^VEGFR2^+^, CD34^+^CD45^dim^VEGFR2^+^)^6,42^ and subsequent cell surface expression of CXCR4 and CXCR7 are displayed in Fig. 1. Selected time-points were run in duplicate, with blood from a single vacutainer separated and fluorescent-labelled antibodies added before analysis by flow cytometry, with an intra-individual CV% of 8.68%.

### Statistical analysis

Statistically significant differences between the type 1 diabetes and non-diabetes control group were determined by independent sample T-test. Data were assessed for normality and outliers by box-plots and Shapiro–Wilk test. Excessively skewed data were transformed using square root and logarithmic transformation. When transformation failed, group difference data were assessed by Mann–Whitney U Test. Time course change data (pre, immediately post and 1 h post exercise) was analysed by mixed-effects model. GraphPad Prism 8.0.1 (San Diego, USA) and IBM SPSS Statistics (version 24, IBM, Armonk NY) software packages were used to analyse the data. Statistical significance set at *p* ≤ 0.05. Data are presented as mean ± standard deviation throughout.

### Ethics approval

All participants provided written informed consent and the study was approved by the NHS HRA North East Tyne & Wear South Research Ethics and Newcastle University Ethics Committees (code:16/NE/0192).

## Results

Demographic data are shown in Table 1. Age, BMI and $\dot{V}{\text{O}}_{{2{\text{peak}}}}$ were comparable between the matched groups.

**Table 1:** Participant demographic data.

	Type 1 diabetes group	Non-diabetes control group	*p*-value
N	30	30	
Male/female	16/14	16/14	
Age (years)	38.2 ± 12.0	37.6 ± 12.1	0.840
HBA1C (mmol/mol)	58.5 ± 9.1	33.5 ± 2.3	**< 0.001**
(%)	7.5 ± 3.0	5.2 ± 2.4	**< 0.001**
BMI (kg/m^2^)	25.2 ± 3.7	24.7 ± 4.6	0.656
$\dot{V}{\text{O}}_{{2{\text{peak}}}}$ (ml/kg/min)	38.8 ± 9.5	42.4 ± 12.4	0.205
Age at diagnosis	18.2 ± 8.6	–	
Range (years)	8 to 35		
Duration of diabetes	20.0 ± 13.0	–	
Range (years)	3 to 47		
Method of control (MDI/CSII)	15/15	–	

Bold signifies *p* ≤ 0.05.

Data presented as mean ± SD. *P* value from independent samples t-test.

On average, participants exercised at 58.8% of their $\dot{V}{\text{O}}_{{2{\text{peak}}}}$, with no differences between the groups (*p* = 0.907). There were no episodes of hypoglycemia (< 3.9 mmol/L) during the exercise bout.

### Resting levels of circulating HPCs and EPCs are lower in the participants with type 1 diabetes than non-diabetes controls

**Figure 2:**
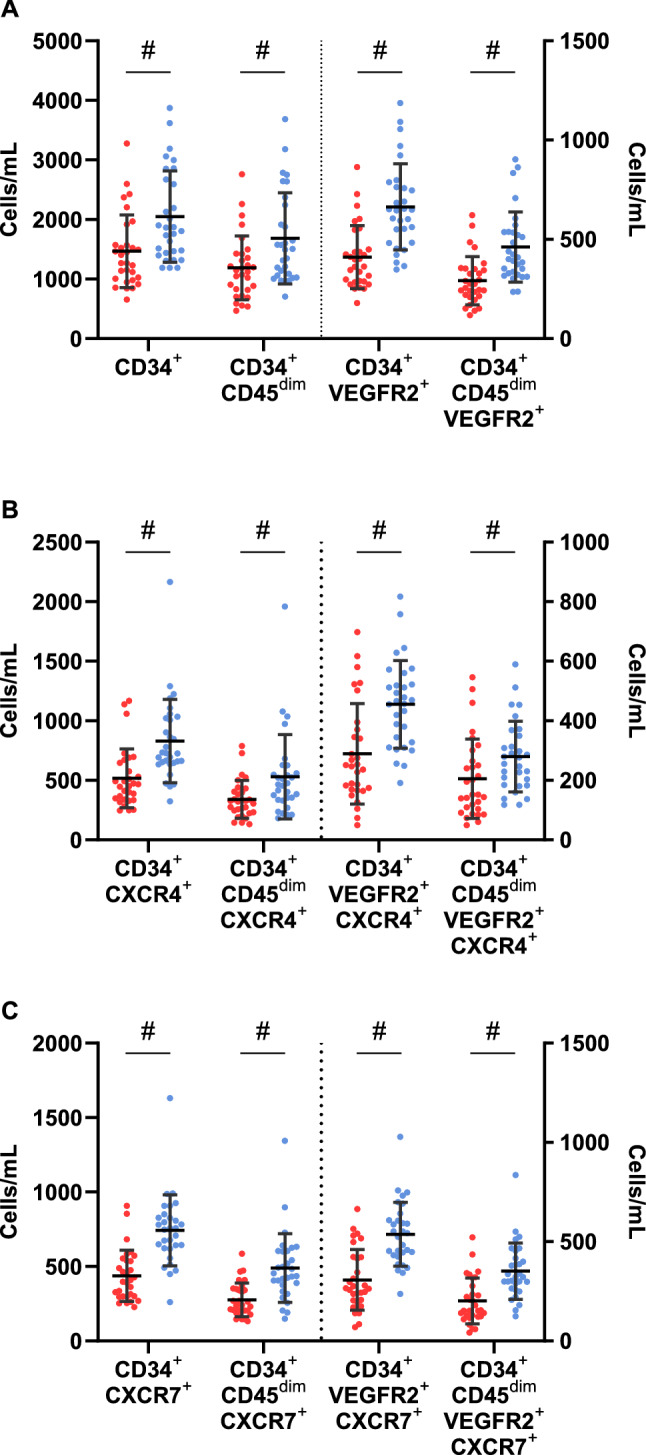
Resting circulating number of CD34^+^, CD34^+^CD45^dim^ HPCs and CD34^+^VEGFR2^+^, CD34^+^CD45^dim^VEGFR2^+^ EPCs (**A**), and the number of these cells expressing CXCR4^+^ (**B**) and CXCR7^+^ (**C**) between the type 1 diabetes (red circles) and non-diabetes (blue circles) groups. #—signifies significant difference between the type 1 diabetes and non-diabetes groups. Data shown are mean ± SD.

Circulating numbers of HPCs CD34^+^ (type 1 diabetes; 1468 ± 611 cells/mL, CON; 2048 ± 768 cells/mL, *p* = 0.001) and CD34^+^CD45^dim^ (type 1 diabetes; 1189 ± 536 cells/mL, CON; 1684 ± 765 cells/mL, *p* = 0.003) were significantly lower at rest in the type 1 diabetes group compared to the non-diabetes controls (Fig. 2A). Resting counts of EPCs CD34^+^VEGFR2^+^ (type 1 diabetes; 411 ± 159 cells/mL, CON; 664 ± 217 cells/mL, *p* < 0.001) and CD34^+^CD45^dim^VEGFR2^+^ (type 1 diabetes; 292 ± 121 cells/mL CON; 462 ± 177 cells/mL, *p* < 0.001) were also significantly lower at rest within the type 1 diabetes group compared to the non-diabetes controls (Fig. 2A). Additionally, circulating number of all HPCs and EPCs expressing CXCR4 and CXCR7 were significantly lower in the type 1 diabetes group than the matched non-diabetes controls (Fig. 2B,C).

**Figure 3:**
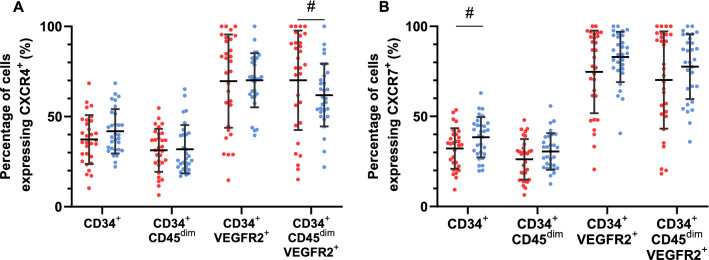
The percentage of CD34^+^, CD34^+^CD45^dim^ HPCs and CD34^+^VEGFR2^+^, CD34^+^CD45^dim^VEGFR2^+^ EPCs expressing CXCR4^+^ (**A**) and CXCR7^+^ (**B**) between the type 1 diabetes (red circles) and non-diabetes (blue circles) groups. #—signifies significant difference between the type 1 diabetes and non-diabetes groups. Data shown are mean ± SD.

When expressed as a percentage of the HPC and EPC phenotypes, CXCR4 expression at rest tended to be similar between groups. However, percentage of CD34^+^CD45^dim^VEGFR2^+^ expressing CXCR4 was significantly higher in in the type 1 diabetes group (*p* = 0.050) (Fig. 3A). Percentage of cells expressing CXCR7 at rest tended to be lower in the type 1 diabetes group, with CD34^+^CXCR7^+^ significantly so (*p* = 0.035) (Fig. 3B).

### Type 1 diabetes patients display attenuated HPC and EPC mobilization in response to acute exercise

**Figure 4:**
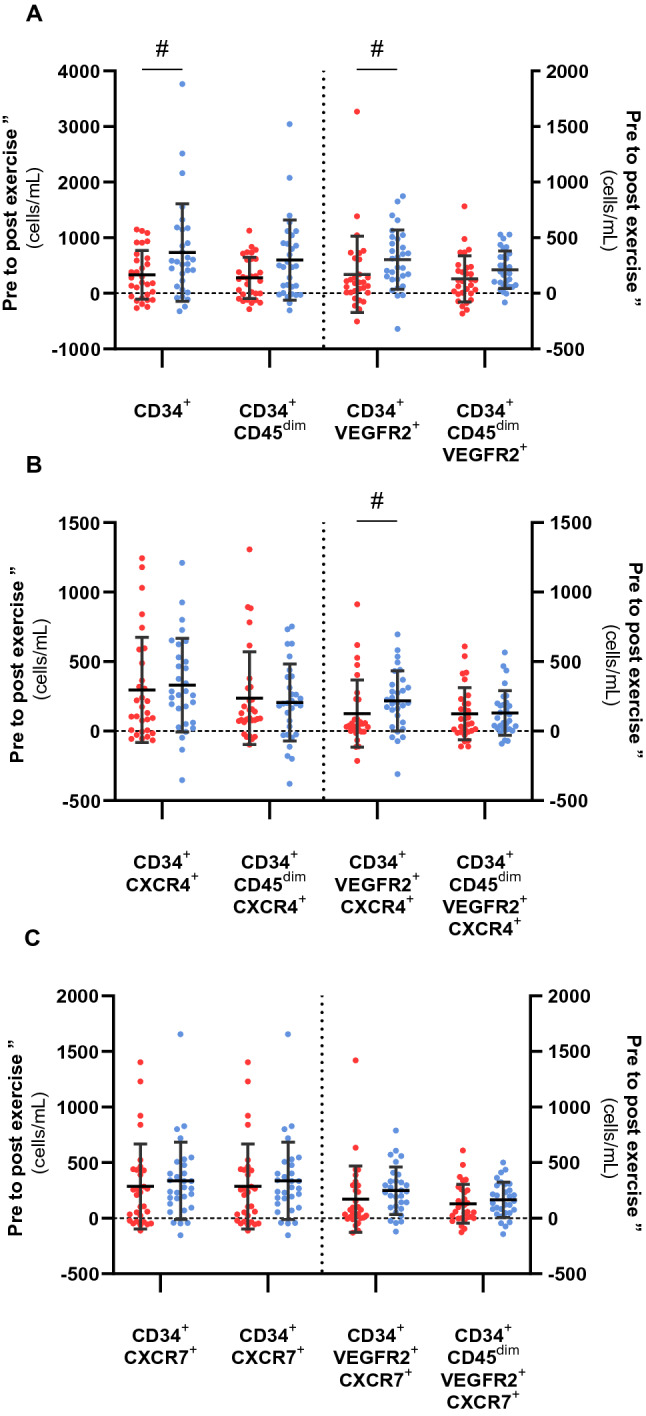
Pre to post exercise delta change (∆ cells, cells/mL) of HPCs and EPCs (**A**), HPCs and EPCs expressing CXCR4^+^ (**B**), HPCs and EPCs expressing CXCR7^+^ (**C**) in participants with type 1 diabetes (red circle) and non-diabetes controls (blue circle) in response to a single bout of moderate-intensity exercise. #—signifies significant difference between the type 1 diabetes and non-diabetes groups. Data shown are mean ± SD.

The mean delta change (Δ) in pre to post-exercise cell numbers is displayed in Fig. 4. The type 1 diabetes group had attenuated mobilization of HPCs and EPCs, ranging from 39 to 55% lower across the phenotypes when compare to the non-diabetes group, with CD34^+^ HPCs (331 ± 437 Δ cells/mL vs. 734 ± 876, Δ cells/mL *p* = 0.048) and CD34^+^VEGFR2^+^ EPCs (171 ± 342 Δ cells/mL vs. 303 ± 267 Δ cells/mL, *p* = 0.006) significantly lower.

There were no significant differences between the groups in the Δ of CXCR4^+^ or CXCR7^+^ HPC and EPC phenotypes (*p* > 0.05), other than for CD34^+^VEGFR2^+^CXCR4^+^ EPCs, where the mobilization in the type 1 diabetes group was 42% lower compared to controls (126 ± 242 Δ cells/mL vs. 218 ± 217 Δ cells/mL, *p* = 0.040).

Within the type 1 diabetes group, exercised-induced increases of the HPCs and EPCs was significantly greater in the cells that also expressed CXCR4 or CXCR7, with the progenitor cells negative for a chemokine receptor having between 64 and 101% less mobilization (Table 2). In comparison, within the controls, only the CD34^+^VEGFR2^+^ EPCs positive for CXCR4 had significantly higher mobilization than the CXCR4 negative cells (218 ± 217 Δ cell/mL vs. 85 ± 143 Δ cell/mL, *p* = 0.007). Additionally, the CD34^+^VEGFR2^+^ and CD34^+^CD45^dim^VEGFR2^+^ EPCs positive for CXCR7 also had significantly greater mobilization than those negative for CXCR7 (248 ± 213 Δ cell/mL vs. 55 ± 132 Δ cell/mL, *p* < 0.001 and 166 ± 158 Δ cell/mL vs. 46 ± 112 Δ cell/mL, *p* = 0.005, respectively).

**Table 2:** Mean delta change (Δ) in pre to post-exercise cell numbers of HPCs and EPCs expressing CXCR4 and CXCR7 versus those negative for CXCR4 and CXCR7 for the type 1 diabetes and control groups.

	CXCR4^+^	CXCR4^−^	*p*	CXCR7^+^	CXCR7^−^	*p*
**Type 1 diabetes group**						
CD34^+^	297 ± 378	34 ± 268	**0.006**	286 ± 383	45 ± 293	**0.018**
CD34^+^CD45^dim^	237 ± 333	40 ± 267	**0.031**	203 ± 283	74 ± 279	0.105
CD34^+^VEGFR2^+^	126 ± 242	44 ± 178	0.084	171 ± 298	− 1 ± 85	**0.002**
CD34^+^CD45^dim^VEGFR2^+^	124 ± 186	5 ± 75	**0.003**	130 ± 175	− 1 ± 75	**< 0.001**
**Control group**						
CD34^+^	332 ± 337	403 ± 641	0.468	337 ± 348	397 ± 766	0.686
CD34^+^CD45^dim^	206 ± 278	391 ± 631	0.173	227 ± 243	380 ± 631	0.311
CD34^+^VEGFR2^+^	218 ± 217	85 ± 143	**0.007**	248 ± 213	55 ± 132	**< 0.001**
CD34^+^CD45^dim^VEGFR2^+^	130 ± 161	82 ± 131	0.276	166 ± 158	46 ± 112	**0.005**

Bold signifies *p* ≤ 0.05.

Data presented as mean ± SD. *P* value from dependent samples t-test.

### Time course kinetics and association between clinical variables and resting and exercise-induced progenitor cell number

All HPC and EPC phenotypes, and their cell surface expression of CXCR4 and CXCR7, had a main effect of time with immediately post-exercise sample significantly higher than the baseline samples (*p* < 0.002). Additionally, CD34^+^, CD34^+^CXCR4^+^, CD34^+^CXCR7^+^ HPCs had a significantly higher count 1-h post-exercise compared to pre-exercise levels (*p* = 0.042, *p* = 0.010 and *p* = 0.013, respectively). There was a group x time interaction for the CD34^+^ HPCs, remaining elevated at 1 h post exercise in the type 1 diabetes group but not the healthy controls (Supplementary Fig. 1).

Clinical variables (HbA1c, BMI, age, $\dot{V}{\text{O}}_{{2{\text{peak}}}}$, age at diagnosis and duration of diabetes) were assessed for correlations with resting concentrations and Δ from pre- to post-exercise (cells/mL) (Supplementary Table 1, 2). HbA1c was negatively correlated with HPC and EPC concentration at rest for all participants (n = 60, *r* > − 0.272, *p* < 0.036). However, when split into the type 1 diabetes (n = 30) and non-diabetes control (n = 30) groups, the relationships were no longer significant, except for HbA1c and CD34^+^CD45^dim^VEGFR2^+^CXCR7^+^ EPCs (*r* = − 0.364, *p* = 0.048) in the type 1 diabetes group. An older age of type 1 diabetes diagnosis positively correlated with CD34^+^CD45^dim^ cells (*r* = 0.361, *p* = 0.050). Within the type 1 diabetes group, no clinical variable correlated with Δ in HPCs or EPCs from pre- to post-exercise.

## Discussion

We investigated the influence of type 1 diabetes on circulating HPC and EPC numbers, and the cell surface expression of CXCR4 and CXCR7 on these cells, at rest and in response to a submaximal exercise bout. For the first time, we demonstrate that individuals with type 1 diabetes are able to increase HPCs and EPCs into circulation in response to exercise. However, mobilization of these angiogenic cells is attenuated in comparison to matched non-diabetes controls, which may play a role in the increased risk of vascular complications seen in type 1 diabetes.

Our primary finding that individuals with type 1 diabetes can mobilize HPCs and EPCs in response to exercise is of interest, as exercise-induced mobilization has been shown to be a more powerful predictor of complications and mortality than basal circulating count in thoracic surgery and coronary artery disease patients^27,28^, and contrasts previous research which found no mobilisation of EPCs in type 1^35,36^ or 2 diabetes^26^. Differences between our study and those previously exploring exercise-induced mobilisation in people with type 1 diabetes likely arose due to alternative ways of quantifying circulating angiogenic cell numbers. While our study used BD Trucount tubes to calculate absolute cell counts and adjusted these results for changes in blood volume^41^, accurately determining cell changes in response to an exercise stressor, previous studies have only measured circulating EPCs as a percentage of circulating mononuclear cells, where any exercise-induced mobilization was likely concealed by increases in overall leucocyte counts around exercise^37^. Additionally, we included a much deeper examination of angiogenic cell phenotypes, demonstrating that both HPCs and EPCs are mobilised by individuals with type 1 diabetes.

These results again demonstrate that type 1 diabetes has a detrimental impact on circulating EPCs and HPCs, with previous research demonstrating a reduced resting count^10–12^ and impaired angiogenic function including: impaired ability to differentiate into endothelial cells, reduced migration to areas of ischemia, reduced angiogenic paracrine secretion, and increased apoptosis^43^. As these circulating cells play an important role in maintaining endothelial integrity^7^, the reduced circulating numbers seen in this study may play an important causative role in the development of diabetic complications and increased CVD through reduced endothelial repair^8^, with lower levels of both HPCs and EPCs counts associated with extensive multi-site atherosclerosis^44^. Our study is the first to demonstrate that circulating numbers of these angiogenic cells expressing CXCR4 and CXCR7 are also significantly lower in individuals with type 1 diabetes, findings similar to those seen in people with type 2 diabetes^45^. The reduced number of cells expressing CXCR4 and CXCR7 likely results in the reduced ability to migrate into circulation and to ischemic tissue within diabetes^29,30,46^, which may further exacerbate endothelial dysfunction and microvascular abnormalities and increase the risk of mortality^47^.

Within our study, both groups were well matched, the participants were not obese or old, and had moderate cardiorespiratory fitness (38.8 ± 9.5 mL/min/kg), which contrasts enormously to work conducted exploring exercise-induced mobilisation of EPCs in type 2 diabetes^26^. Additionally, our participants with type 1 diabetes had no major diabetes-related complications. Despite this, we showed that the increased circulating HPCs and EPCs from pre- to post-exercise in the type 1 diabetes group, CD34^+^ HPCs, CD34^+^VEGFR2^+^, CD34^+^VEGFR2^+^CXCR4^+^ EPC counts were significantly attenuated compared to the non-diabetes controls. Strikingly, mean post-exercise concentrations of most the phenotypes were lower in the type 1 diabetes group than the resting concentrations of the controls. The reduced exercise-induced mobilization is similar to previous studies that found no mobilisation of HPCs and EPCs to indirect CXCR4^+^ stimulation^31^ and slightly attenuated mobilisation to direct CXCR4^+^ antagonists in a mixed group of type 1 and 2 diabetes participants^32^. It is unclear why a direct CXCR4^+^ antagonist can mobilise angiogenic cells from the bone marrow while an indirect cannot. As exercise mobilised HPCs and EPCs negative for CXCR4 and CXCR7 in the controls, but not the type 1 diabetes group, this suggests pathways other than stromal cell–derived factor-1α (SDF-1α)/CXCR4 are also impaired by deregulated glucose control seen in diabetes.

While the exact mechanism for mobilising angiogenic cells in response to exercise has not been fully elucidated, mobilization is dependent on both duration and intensity, with a higher intensity potentially needed in this study in order for all participants to achieve mobilisation^23^. Post-exercise counts have been shown to positively correlate with increased circulating levels of SDF-1α, VEGF, erythropoietin and tissue expression of hypoxia-inducible factor 1-α. Suppressed release of VEGF and SDF-1α, key for the mobilization and homing of progenitor cells from the bone marrow to areas of ischemia, have been demonstrated in a murine model of diabetes^48^, and may explain the reduced increase in HPCs and EPCs seen in this study. Additionally, high glucose conditions have been shown to reduce the angiogenic function of HPCs and EPCs^49^, as well as increasing senescence and apoptosis^43^. Within type 1 diabetes mouse models, it has been demonstrated that increased vascular damage ultimately results in the exhaustion and depletion of progenitor cells stored within the bone marrow. Moreover, dysfunctional osteoblastic niches and microangiopathy damage to the blood vessels in the bone resulting in an impaired ability to egress these cells into circulation in response to ischemia^50^. Microvascular dysfunction and altered blood flow can occur in the early stages of Type 1 diabetes, with hyperglycaemia and oxidative stress reducing the bioavailabilty of nitrix oxide^51,52^. Endothelial nitrix oxide synthase induces smooth muscle relaxition and blood vessel dilation, and is strong modulator of circulating angiogenic cells funtion and homing^53^. It has been demonstrated that pancreas transplants improves endothelial function in conjunction with the normalisation of glucose metabolism by restoring endothelial nitric oxide synthase^54^, which likely explains the post islet-transplant improvements in circulating angiogenic cell function^55^. This raises the intriguing possibility that exercise, improvements in glycemic control and vasodilatory dietary supplements could increase endothelial nitric oxide synthase, improving endothelial and angiogenic cells functions within people with type 1 diabetes, warranting further study^56^.

There is growing evidence for separate and important functions of CXCR7, promoting endothelial proliferation and angiogenesis, and plying a critical role in the survival of EPCs. Our results are supported by the observations by Dai et al.^30^ demonstrating that the percentage of EPCs expressing CXCR7 but not CXCR4 was reduced in a diabetes mouse and in vitro model, but not Vigorelli et al.^57^ who showed reduced CXCR4 protein expression when exposing CD34^+^ cells to a high glucose environment in vitro. As knockdown of CXCR7 impairs vascular tube formation and upregulation rescues angiogenic function of diabetic EPCs^30^, the reduction in CXCR7 angiogenic cells within this study is of clinical significance, highlighting the dysfunctional nature of these cells in people with type 1 diabetes. The effect of glucose upon CXCR4 is controversial, with high glucose reported to both increase^58^ and inhibit expression^59^.

Limitations of this study include the lack of an apoptosis marker, making it likely that non-viable cells were quantified. This especially true of EPCs within the type 1 diabetes group, where increased apoptosis is likely due to hyperglycemia^43^, and mean fluorescence intensity of VEGFR2 staining is slightly greater in dead versus live cells^60^. Measuring progenitor cell mobilising stimuli would also have been beneficial, especially as the number of HPCs negative for a chemokine receptor mobilized with exercise was substantially lower in the type 1 diabetes group suggesting impairment of an additional pathway other than the SDF-1α/CXCR4 axis. Future research needs to explore whether different methods of diabetes management and improving glycemic control results in improvement in exercise-induced mobilization of these cells within individuals with type 1 diabetes. Improving HbA1c, and reducing glycemic variability (by switching diabetes management to continuous subcutaneous insulin infusion) both increase basal concentrations of EPCs^13,61^, while severe hypoglycemia is associated with a marked depletion of circulating HPCs and EPCs in individuals with type 2 diabetes^62^. Potentially, they also influence exercise-induced mobilization. Regular exercise training, in both healthy and diseased populations, has also been shown to increase basal concentration of EPCs and HPCs^62^. Therefore, determining if exercise training could increase basal concentration and restore exercise-induced mobilization in individuals with type 1 diabetes, with the aim of improving vascular repair and reducing both micro and macrovascular diabetes complications merits further study.

## Conclusion

In conclusion, people with type 1 diabetes have reduced resting and attenuated mobilization of EPCs and HPCs with exercise compared to matched controls. Reduced mobilization of HPCs and EPCs with exercise may play a role in the increased cardiovascular risk in individuals with type 1 diabetes.

## Supplementary Information

Supplementary Information.

## Data Availability

The datasets used during the current study are available from the corresponding author (Daniel J West; Email: daniel.west@newcastle.ac.uk, telephone: + 44 (0)191 20 87076) on reasonable request.

## Abbreviations

CVD: Cardiovascular diseases
CXCR4: Chemokine receptor 4
CXCR7: Chemokine receptor 7
EPCs: Endothelial progenitor cells
HPCs: Haematopoietic progenitor cells
CRF: Newcastle NIHR Clinical Research Facility
