# Supplementary material for: Type 1 diabetes patients increase CXCR4+ and CXCR7+ haematopoietic and endothelial progenitor cells with exercise, but the response is attenuated
Source: Sci Rep. 2021 Jul 15;11:14502. doi: 10.1038/s41598-021-93886-2 (PMC8282661; doi:10.1038/s41598-021-93886-2)
Supplement: Supplementary file 1 — Supplementary Information. [file 41598_2021_93886_MOESM1_ESM.docx]

Type 1 diabetes patients can mobilise CXCR4^+^ and CXCR7^+^ haematopoietic and endothelial progenitor cells with exercise, but the response is attenuated.

Short title:

Exercise mobilization of HPCs and EPCs in T1D

Authors:

Guy S Taylor^1^ PhD, Andy Shaw^1^ MBBS, Kieran Smith^1^ MSc, Tess E Capper^1,3^ PhD, Jadine H Scragg^14^ MSci, Michael Cronin^6^ MRes, Ayat Bashir^2^ MRCP, Anneliese Flatt^2^ MRCP, Matthew D Campbell^56^ PhD, Emma J Stevenson^1^ PhD, James A Shaw^2^ PhD, Mark Ross^7^ PhD, Daniel J West^1^ PhD.

1 Population Health Sciences Institute, Newcastle University, Newcastle upon Tyne, UK

2 Translational and Clinical Research Institute, Newcastle University, Newcastle upon Tyne, UK

3 Centre for Public Health, Queen’s University Belfast, Belfast, UK

4 Nuffield Department of Primary Care Health Sciences, University of Oxford, Oxford, UK

5 Faculty of Health Sciences and Wellbeing, University of Sunderland, Sunderland, UK

6 Leeds Institute of Cardiovascular and Metabolic Medicine, University of Leeds, Leeds, UK

7 School of Applied Sciences, Edinburgh Napier University, Edinburgh, UK.

**
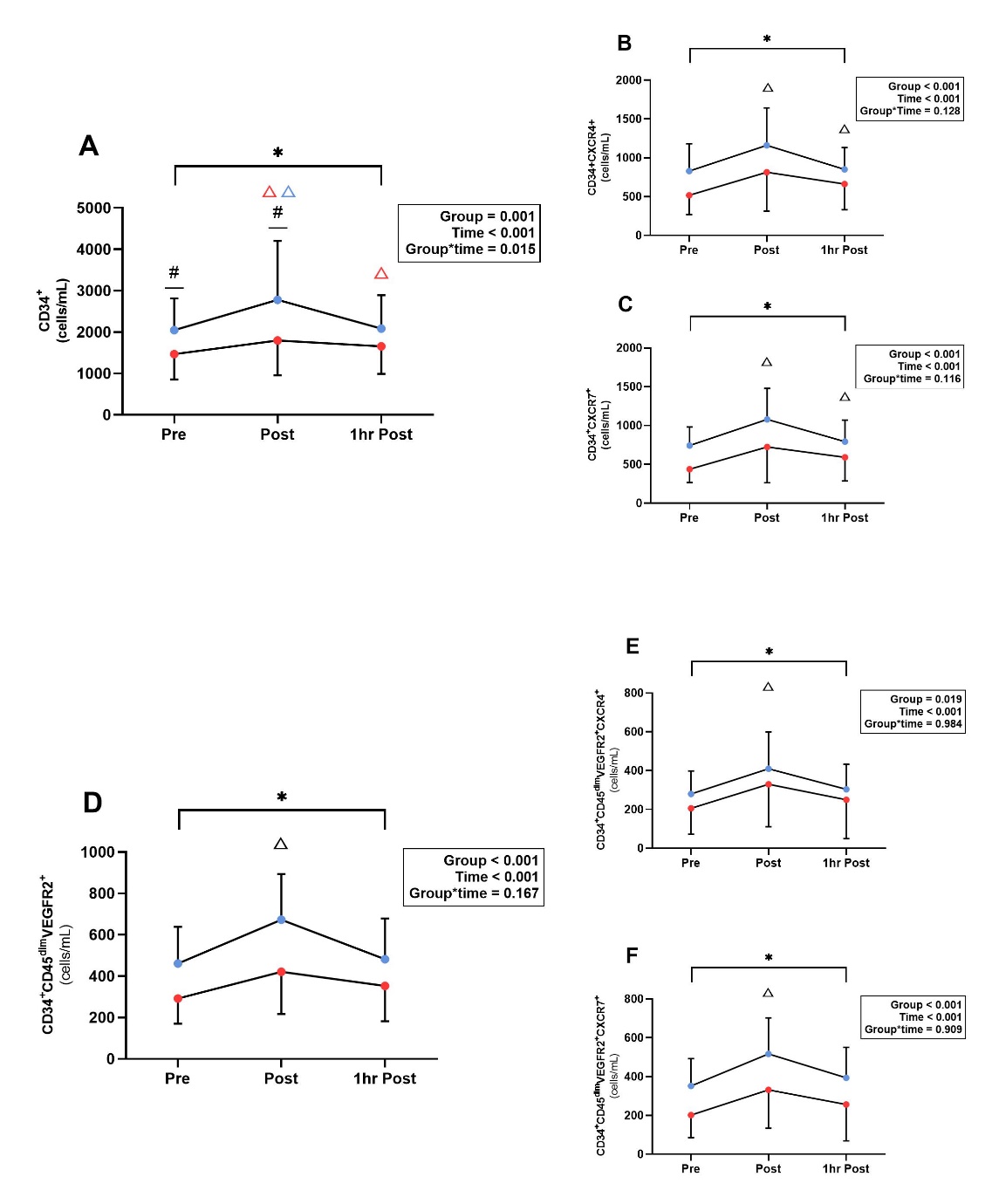
**

**Supplementary Figure 1.** Exercise-induced mobilization of HPCs in participants with type 1 diabetes (red circle) and non-diabetes controls (blue circle). Absolute count (cells/mL) of HPCs; CD34+ (A), CD34+CXCR4+ (B) and CD34+CXCR7+ (C) and EPCs; CD34+CD45dimVEGFR2+ (D), CD34+CD45dimVEGFR2+CXCR4+ (E), CD34+CD45dimVEGFR2+CXCR7+ (F) cells in response to a single bout of moderate-intensity exercise. * - Significant main effect of group differences, △ – Significant main effect of time difference from baseline, # - Significant group differences at timepoint, △ (red triangle) - Significant time difference from baseline in the type 1 diabetes group, △ (blue triangle) - Significant time difference from baseline in non-diabetes group. *Data presented as mean ± SD.*

**Supplementary Table 1.** Spearman’s rank order correlations assessing clinical variables against resting concentrations of HPC and EPC phenotypes.

|  |  | HbA1c | | BMI | | Age | | VO_2peak_ | | Age at Diagnosis | | Duration of diabetes | |
| --- | --- | --- | --- | --- | --- | --- | --- | --- | --- | --- | --- | --- | --- |
|  |  | r | p | r | p | r | p | r | p | r | p | r | p |
| CD34^+^ | All | **-0.350** | **0.006** | -0.115 | 0.380 | 0.050 | 0.704 | 0.134 | 0.408 | - |  | - |  |
|  | T1D | 0.072 | 0.706 | 0.069 | 0.718 | **0.390** | **0.033** | 0.023 | 0.902 | 0.270 | 0.149 | 0.126 | 0.505 |
|  | Controls | -0.029 | 0.879 | -0.245 | 0.191 | -0.295 | 0.113 | 0.209 | 0.268 | - |  | - |  |
| CD34^+^ CXCR4^+^ | All | **-0.531** | **<0.001** | -0.138 | 0.294 | -0.118 | 0.370 | 0.228 | 0.080 | - |  | - |  |
|  | T1D | -0.266 | 0.156 | 0.015 | 0.938 | 0.120 | 0.528 | 0.131 | 0.489 | 0.040 | 0.833 | 0.159 | 0.401 |
|  | Controls | -0.104 | 0.583 | -0.166 | 0.380 | -0.350 | 0.058 | 0.251 | 0.180 | - |  | - |  |
| CD34^+^ CXCR7^+^ | All | **-0.623** | **<0.001** | -0.125 | 0.343 | -0.101 | 0.441 | 0.219 | 0.093 | - |  | - |  |
|  | T1D | -0.270 | 0.149 | -0.069 | 0.718 | 0.035 | 0.854 | 0.241 | 0.199 | -0.093 | 0.625 | 0.208 | 0.270 |
|  | Controls | -0.241 | 0.200 | -0.017 | 0.928 | -0.233 | 0.215 | 0.051 | 0.788 | - |  | - |  |
| CD34^+^ CD45^dim^ | All | **-0.272** | **0.036** | -0.042 | 0.749 | 0.137 | 0.296 | 0.066 | 0.615 | - |  | - |  |
|  | T1D | 0.146 | 0.443 | 0.077 | 0.685 | **0.495** | **0.005** | -0.036 | 0.850 | **0.361** | **0.050** | 0.101 | 0.595 |
|  | Controls | -0.018 | 0.925 | -0.107 | 0.572 | -0.193 | 0.306 | 0.137 | 0.471 | - |  | - |  |
| CD34^+^ CD45^dim^ CXCR4^+^ | All | **-0.367** | **0.004** | -0.035 | 0.792 | -0.135 | 0.304 | 0.146 | 0.265 | - |  | - |  |
|  | T1D | -0.229 | 0.223 | -0.071 | 0.708 | 0.070 | 0.714 | 0.144 | 0.446 | 0.088 | 0.644 | 0.031 | 0.870 |
|  | Controls | -0.013 | 0.945 | 0.102 | 0.593 | -0.379 | 0.039 | 0.093 | 0.624 | - |  | - |  |
| CD34^+^ CD45^dim^ CXCR7^+^ | All | **-0.573** | **<0.001** | -0.063 | 0.632 | -0.041 | 0.757 | 0.187 | 0.152 | - |  | - |  |
|  | T1D | -0.231 | 0.218 | -0.195 | 0.302 | 0.032 | 0.868 | 0.319 | 0.086 | 0.003 | 0.986 | 0.047 | 0.804 |
|  | Controls | -0.133 | 0.485 | 0.118 | 0.534 | -0.134 | 0.480 | -0.004 | 0.981 | - |  | - |  |
| CD34+ VEGFR2^+^ | All | **-0.557** | **<0.001** | -0.150 | 0.251 | -0.131 | 0.320 | 0.238 | 0.067 | - |  | - |  |
|  | T1D | -0.057 | 0.765 | -0.075 | 0.694 | -0.079 | 0.678 | 0.076 | 0.691 | -0.100 | 0.599 | 0.079 | 0.679 |
|  | Controls | -0.097 | 0.612 | -0.193 | 0.307 | -0.272 | 0.145 | **0.374** | **0.042** | - |  | - |  |
| CD34^+^ VEGFR2^+^ CXCR4^+^ | All | **-0.505** | **<0.001** | -0.123 | 0.348 | -0.118 | 0.367 | **0.298** | **0.021** | - |  | - |  |
|  | T1D | -0.299 | 0.109 | -0.074 | 0.697 | 0.088 | 0.643 | 0.146 | 0.440 | 0.030 | 0.876 | 0.092 | 0.627 |
|  | Controls | 0.003 | 0.986 | -0.076 | 0.689 | -0.335 | 0.70 | **0.453** | **0.012** | - |  | - |  |
| CD34^+^ VEGFR2^+^ CXCR7^+^ | All | **-0.603** | **<0.001** | -0.142 | 0.278 | -0.081 | 0.541 | 0.241 | 0.064 | - |  | - |  |
|  | T1D | -0.313 | 0.092 | -0.008 | 0.966 | 0.097 | 0.611 | 0.053 | 0.780 | -0.027 | 0.886 | 0.183 | 0.333 |
|  | Controls | 0.13 | 0.944 | -0.098 | 0.608 | -0.231 | 0.219 | 0.275 | 0.142 | - |  | - |  |
| CD34^+^  CD45^dim^ VEGFR2^+^ | All | **-0.523** | **<0.001** | -0.120 | 0.362 | -0.099 | 0.450 | 0.157 | 0.231 | - |  | - |  |
|  | T1D | 0.006 | 0.976 | -0.150 | 0.428 | -0.034 | 0.858 | 0.077 | 0.685 | -0.012 | 0.950 | -0.029 | 0.879 |
|  | Controls | -0.243 | 0.196 | -0.101 | 0.596 | -0.215 | 0.254 | 0.192 | 0.310 | - |  | - |  |
| CD34^+^ CD45^dim^ VEGFR2^+^ CXCR4^+^ | All | **-0.403** | **0.001** | -0.061 | 0.641 | -0.133 | 0.310 | 0.233 | 0.074 | - |  | - |  |
|  | T1D | -0.312 | 0.093 | -0.046 | 0.809 | 0.094 | 0.622 | 0.205 | 0.277 | 0.116 | 0.541 | 0.054 | 0.775 |
|  | Controls | -0.118 | 0.536 | -0.010 | 0.958 | **-0.375** | **0.041** | 0.281 | 0.133 | - |  | - |  |
| CD34^+^ CD45^dim^ VEGFR2^+^ CXCR7^+^ | All | **-0.577** | **<0.001** | -0.114 | 0.387 | -0.057 | 0.665 | 0.179 | 0.171 | - |  | - |  |
|  | T1D | **-0.364** | **0.048** | -0.140 | 0.461 | 0.033 | 0.861 | 0.192 | 0.309 | 0.116 | 0.540 | -0.015 | 0.938 |
|  | Controls | -0.125 | 0.512 | 0.010 | 0.960 | -0.173 | 0.361 | 0.078 | 0.683 | - |  | - |  |

**Supplementary Table 2.** Spearman’s rank order correlations assessing clinical variables against delta change in pre to post-exercise cell numbers of HPC and EPC phenotypes.

|  |  | HbA1c | | BMI | | Age | | VO_2peak_ | | Age at Diagnosis | | Duration of diabetes | |
| --- | --- | --- | --- | --- | --- | --- | --- | --- | --- | --- | --- | --- | --- |
|  |  | r | p | r | p | r | p | r | p | r | p | r | p |
| CD34^+^ | All | -0.253 | 0.051 | -0.085 | 0.517 | -0.112 | 0.392 | 0.133 | 0.311 | - |  | - |  |
|  | T1D | 0.022 | 0.907 | -0.275 | 0.141 | -0.072 | 0.707 | 0.047 | 0.804 | 0.244 | 0.194 | -0.096 | 0.615 |
|  | Controls | -0.158 | 0.404 | 0.059 | 0.759 | -0.137 | 0469 | 0.199 | 0.291 | - |  | - |  |
| CD34^+^ CXCR4^+^ | All | -0.125 | 0.342 | -0.034 | 0.796 | -0.078 | 0.551 | 0.092 | 0.487 | - |  | - |  |
|  | T1D | 0.029 | 0.879 | -0.137 | 0.469 | -0.055 | 0.772 | 0.050 | 0.794 | 0.063 | 0.741 | 0.017 | 0.927 |
|  | Controls | -0.092 | 0.630 | 0.062 | 0.746 | -0.100 | 0.597 | 0.174 | 0.358 | - |  | - |  |
| CD34^+^ CXCR7^+^ | All | -0.114 | 0.386 | -0.083 | 0.526 | -0.140 | 0.286 | 0.094 | 0.475 | - |  | - |  |
|  | T1D | -0.069 | 0.718 | -0.116 | 0.540 | -0.069 | 0.717 | -0.010 | 0.956 | -0.084 | 0.659 | 0.187 | 0.323 |
|  | Controls | 0.125 | 0.510 | -0.103 | 0.588 | -0.245 | 0.193 | 0.218 | 0.248 | - |  | - |  |
| CD34^+^ CD45^dim^ | All | -0.224 | 0.085 | -0.060 | 0.651 | -0.104 | 0.429 | 0.143 | 0.274 | - |  | - |  |
|  | T1D | 0.078 | 0.680 | -0.222 | 0.238 | -0.097 | 0.609 | 0.038 | 0.841 | 0.252 | 0.179 | -0.085 | 0.657 |
|  | Controls | -0.203 | 0.283 | 0.086 | 0.651 | -0.071 | 0.708 | 0.185 | 0.327 | - |  | - |  |
| CD34^+^ CD45^dim^ CXCR4^+^ | All | -0.111 | 0.398 | 0.085 | 0.518 | -0.135 | -0.120 | 0.012 | 0.930 | - |  | - |  |
|  | T1D | -0.096 | 0.613 | 0.016 | 0.934 | 0.070 | -0.195 | -0.008 | 0.968 | 0.065 | 0.722 | -0.088 | 0.645 |
|  | Controls | -0.158 | 0.405 | 0.097 | 0.609 | -0.379 | -0.076 | 0.085 | 0.655 | - |  | - |  |
| CD34^+^ CD45^dim^ CXCR7^+^ | All | -0.080 | 0.546 | -0.029 | 0.826 | -0.072 | 0.583 | 0.064 | 0.627 | - |  | - |  |
|  | T1D | -0.191 | 0.312 | 0.072 | 0.706 | -0.111 | 0.560 | -0.038 | 0.841 | 0.129 | 0.498 | -0.085 | 0.655 |
|  | Controls | 0.244 | 0.194 | -0.179 | 0.344 | -0.052 | 0.786 | 0.179 | 0.344 | - |  | - |  |
| CD34+ VEGFR2^+^ | All | **-0.331** | **0.010** | -0.140 | 0.287 | -0.088 | 0.503 | 0.102 | 0.436 | - |  | - |  |
|  | T1D | -0.163 | 0.389 | -0.119 | 0.531 | 0.085 | 0.655 | -0.022 | 0.910 | 0.257 | 0.171 | -0.031 | 0.871 |
|  | Controls | 0.023 | 0.904 | -0.146 | 0.442 | -0.326 | 0.079 | 0.270 | 0.149 | - |  | - |  |
| CD34^+^ VEGFR2^+^ CXCR4^+^ | All | **-0.505** | **<0.001** | -0.151 | 0.250 | -0.032 | 0.807 | 0.010 | 0.942 | - |  | - |  |
|  | T1D | -0.299 | 0.109 | 0.014 | 0.940 | 0.236 | 0.209 | -0.266 | 0.155 | 0.088 | 0.645 | 0.231 | 0.220 |
|  | Controls | 0.003 | 0.986 | -0.231 | 0.220 | -0.272 | 0.146 | 0.193 | 0.307 | - |  | - |  |
| CD34^+^ VEGFR2^+^ CXCR7^+^ | All | **-0.603** | **<0.001** | -0.154 | 0.240 | -0.111 | 0.397 | 0.114 | 0.384 | - |  | - |  |
|  | T1D | -0.313 | 0.092 | -0.143 | 0.449 | 0.009 | 0.964 | -0.015 | 0.939 | 0.211 | 0.264 | -0.117 | 0.536 |
|  | Controls | 0.13 | 0.944 | -0.179 | 0.345 | -0.263 | 0.160 | 0.249 | 0.184 | - |  | - |  |
| CD34^+^  CD45^dim^ VEGFR2^+^ | All | -0.243 | 0.062 | -0.101 | 0.433 | -0.172 | 0.188 | 0.170 | 0.194 | - |  | - |  |
|  | T1D | -0.157 | 0.406 | -0.052 | 0.784 | -0.086 | 0.651 | -0.036 | 0.849 | 0.274 | 0.143 | -0.141 | 0.458 |
|  | Controls | 0.053 | 0.781 | -0.117 | 0.537 | -0.252 | 0.179 | **0.361** | **0.050** | - |  | - |  |
| CD34^+^ CD45^dim^ VEGFR2^+^ CXCR4^+^ | All | -0.076 | 0.566 | -0.058 | 0.657 | -0.147 | 0.261 | 0.018 | 0.891 | - |  | - |  |
|  | T1D | 0.010 | 0.957 | 0.001 | 0.997 | -0.085 | 0.655 | -0.119 | 0.531 | 0.094 | 0.620 | -0.023 | 0.902 |
|  | Controls | -0.015 | 0.937 | -0.094 | 0.623 | -0.193 | 0.307 | 0.131 | 0.489 | - |  | - |  |
| CD34^+^ CD45^dim^ VEGFR2^+^ CXCR7^+^ | All | -0.069 | 0.599 | -0.096 | 0.468 | -0.112 | 0.394 | 0.127 | 0.334 | - |  | - |  |
|  | T1D | -0.121 | 0.523 | 0.006 | 0.973 | -0.099 | 0.602 | -0.071 | 0.707 | 0.159 | 0.403 | -0.123 | 0.517 |
|  | Controls | **0.363** | **0.048** | -0.156 | 0.409 | -0.089 | 0.639 | 0.271 | 0.147 | - |  | - |  |
